# Prevalence and factors associated with road traffic incident among adolescents and children in the hospitals of Amhara National Regional State, Ethiopia

**DOI:** 10.1186/s12873-019-0238-1

**Published:** 2019-03-04

**Authors:** Bewket Tadesse Tiruneh, Berhanu Boru Bifftu, Berihun Assefa Dachew

**Affiliations:** 1 College of Medicine and Health Science, School of Nursing, University of Gondar, P.O.Box: 196, Gondar, Ethiopia; 2 Department of Epidemiology and Biostatistics, Institute of Public Health, College of Medicine and Health Sciences, University of Gondar, P.O.Box: 196, Gondar, Ethiopia

**Keywords:** Adolescent, Children, Factors, Prevalence, Road Traffic Incident

## Abstract

**Background:**

Road Traffic Incident (RTI) has been commonly reported as a major public health problem around the world and the incidence is higher in low and middle income countries, such as Ethiopia than high income countries. The aim of this study was to assess the prevalence and factors associated with RTI among adolescents and children in the hospitals of Amhara Regional State, Ethiopia.

**Methods:**

A cross-sectional study design was employed among 830 injured children visiting the Emergency Department of four randomly selected hospitals of Amhara Regional State, Ethiopia between February 1 and April 30, 2016. Data were entered into EPI info version 7 and then exported to SPSS version 20, for further analysis. Univariable and multivariable logistic regression models were fitted. Adjusted odds ratio with its 95% confidence interval was used to determine the statistical significance.

**Results:**

The overall prevalence of RTI, from all injury cases, was 20%. Socioeconomic status, being wealthy (AOR: 0.08, 95% CI, 0.01, 0.45) and middle income (AOR: 0.40,95% CI, 0.17, 0.97), parental/guardian education/no education (AOR: 6.91, 95% CI, 2.52, 8.93), mothers marital status/divorced (AOR: 0.01, 95% CI, 0.01, 0.05), and leaving a child with another child for sometime (AOR: 2.56, CI 1.06, 6.20) and most often (AOR: 4.77, CI, 1.15, 9.77) were factors independently associated with RTI .

**Conclusion and recommendation:**

The prevalence of RTI was found to be high. RTI prevention needs critical consideration and the intervention strategies shall focus on those families who are practicing of leaving a child with another child.

## Background

Nations of the world have been implementing different strategies towards Road Traffic Incident (RTI), which encompass improving the road system, limiting the speed, enforcement to use seat belts, drink-driving laws, prohibition for phoning during driving and wearing helmets [1]. However, RTI is still continuing as one of the major public health concern and estimated to be one of leading cause of death in the year 2030 [2]. Furthermore, the sequels of being injured in RTI can result in mental and psychological health problems [3].

Globally, the pediatric age population accounted for 21% of RTI related deaths. Data in Great Britain in 2011 showed that RTI was responsible for the death or injury of 2412 children under the age of 16 years. RTI is the leading cause of disability and death for children in Europe, even with a strives to prevent the problem, and create an environment where no child death due to RTI [4]. Data suggests that 93% of child road traffic incident deaths occur in low and middle-income countries [5].

Studies revealed that RTI showed a significant rise in Africa in 2014. For example, the death rate for Kenyans was 19.0 persons per 100,000, while the Ethiopians was 37.3persons per 100,000 in 2014 [6].The road users, the environment, and the condition of the vehicles were factors associated with RTI in Ethiopia. In the year 2005, 93% of the accidents were accounted for road users, and the rest 5 and 2% were accounted for road environment and vehicle related factors respectively. The capital city of Ethiopia, Addis Ababa, and the Oromia National Regional State accounted for nearly 60% of RTI in Ethiopia [3].The government of Ethiopia introduced a number of strategies towards reduction of RTI, which includes safety awareness program, with further scale up of a campaign, improving visibility of road users, following the compliance of drivers with road safety rules [7]. However, RTI is still a common cause of morbidity and mortality in Ethiopia. There is paucity of data on RTI in Ethiopia and to the best of the authors’ knowledge no study among children [8]. Having data on the burden of RTI and identifying the associated factors would help policy-makers and program managers in developing responses for the prevention of RTI. Therefore, this study was designed to determine the prevalence of RTI and to identify factors associated with among children in Amhara National Regional State, Ethiopia.

## Methods

This study was a cross-sectional study design, conducted in four selected hospitals of Amhara National Regional State, Ethiopia (University of Gondar Hospital, Felegehiwot Hospital, Debark Hospital, and Debertabour Hospital), from February 1 to April 30, 2016.These hospitals were randomly selected and included in the study. In this study, all children under the age of 18 visiting the Emergency Department(ED) of these hospitals, due to any forms of injury, was included. Systematic random sampling techniques were used to select study participants in the ED of each hospital. A single population proportion formula was used to determine the sample size and the following assumptions made: 95% confidence level, 5% margin of error and taking prevalence of 50%. Considering a 10% non-response rate, the sample size becomes 423. Taking in to account design effect of two, the final sample size was 846.

Data were collected using an interviewer-administered questionnaire which was prepared by reviewing different studies conducted on injury, including RTI [9–11]. To maintain data quality, training was given to data collectors and supervisors. In addition, supervision was carried out on daily basis during the data collection to check completeness and consistency of the data, both by the supervisors and investigators. The collected data was entered and cleaned using EPI-INFO and analyzed using SPSS version 20. Frequency distributions and percentages were calculated to describe socio-demographic characteristics. Simple and multivariable logistic regression analyses were used to explore associations between RTI and dependent variables. Odds ratios and 95% confidence intervals were used as measures of association. A *p*-value less than < 0.05 in the multivariable model were accepted as statistically significant.

## Results

### Socio-demographic characteristics of respondents

Out of 846 study participants, 830 were participated in the study, making the response rate 98%. Over 60% of them were male, more than a quarter of them were not in school and nearly 16% of the participants were under the age of 5, and nearly 49% of the families were not educated. More than 46% of children’s fathers were farmers by occupation, while majorities (61.2%) of children’s mothers were housewife. Nearly 44%of the children lived with both parents (Table 1).

**Table 1:** Socio-demographic characteristics of study participants, Amhara National, Regional State Referral Hospitals Ethiopia, 2016 (*n* = 830)

Variables		Frequency (%)
Age of parents/guardians in years	50–64	58(7.0)
	35—49	168 (20.2)
	< 30	273 (32.9)
	25–34	331 (39.9)
Sex of children	Female	322 (38.8)
	Male	508 (61.2)
Age of children	< 5	132 (15.9)
	5–10	281 (33.9)
	11–17	417 (50.2)
Children live with	Guardian	149 (18)
	Father or mother	318 (38.3)
	Both parents	363 (43.7)
Occupation of the mother	Day laborer	30 (3.6)
	Employee	126 (15.2)
	Petty trader	163 (19.6)
	Housewife	511 (61.6)
Occupation of the father	Farmer	385 (46.4)
	Employ	235 (28.3)
	Petty trader	88 (10.6)
	Day laborer	122 (14.7)
Marital status of the mother	Divorced	23 (2.8)
	Separated	26 (3.1)
	Single	56 (6.7)
	Widow	71 (8.6)
	Married	654 (78.8)
Marital status of the father	Divorced	25 (3.0)
	Separated	39 (4.7)
	Widow	47 (5.7)
	Single	58(7.0)
	Married	661 (79.6)
Educational status of the parents or guardians	College education	95 (11.4)
	Second education	153 (18.4)
	Primary education	179 (21.6)
	No education	403 (48.6)
Child educational statues	College	6 (0.7)
	Nursery	51 (6.1)
	Secondary	125 (15.1)
	No education	216 (26.0)
	Primary	432 (52.0)
Income description of the parents/guardians	Wealthy	53 (6.4)
	Low	271 (32.7)
	Middle	506 (61.0)

### Prevalence and factors associated with RTI among adolescents and children

In this study, the prevalence of RTI was found to be 20% (95% CI: 17.2, 22.9). From this, 113(68.1%) of the victims were male, 86(51.8%) of them were found in the age group of 11–17. For 121(27.1%) of the cases, cars were involved, and for 92(55.4%) of the victims RTI were experienced while crossing the road. Nearly 70% of the cases arrived in the emergency department immediately (Table 2).

**Table 2:** Prevalence of RTI among Adolescents and Children in the Referral Hospitals of Amhara Regional State, Ethiopia. 2016 (*n* = 166)

Characteristics		Frequency (%)
Sex	Male	113 (68.1)
	Female	53 (31.9)
Age of children in years	Less than 5	22 (13.3)
	6–10	58 (34.9)
	11–17	86 (51.8)
Age of guardian or parents	< 30	50 (30.1)
	31–40	68 (41.0)
	41–50	39 (23.5)
	51–60	9 (5.4)
Type of vehicle involved	Car	121 (27.1)
	Tri -motorcycle	45 (27.1)
Activity at the time of injury	Passenger	38 (22.9)
	While walking along the road	36 (21.7)
	While crossing the road	92 (55.4)
Time of arrival at the Emergency Department	Immediately	115 (69.3)
	Within hours	17 (10.2)
	Within a day	31 (18.7)
	Within days	3 (1.8)
Educational level of the child	No education	40 (24)
	Nursery	5 (3.0)
	Primary	97 (58.4)
	Secondary	24 (14.5)
Mother’s marital status	Single	13 (7.8)
	Married	130 (78.3)
	Separated	3 (1.8%
	Divorced	11 (6.6)
	Widowed	9 (5.4)
Father’s marital status	Single	16 (9.6)
	Married	129 (77.7)
	Separated	2 (1.2)
	Divorced	11 (6.6)
	Widowed	8 (4.8)

### Factors associated with RTI among children

In the multivariable analysis; on socioeconomic status, being wealthy (Adjusted Odd Ratios (AOR):0.08, 95% Confidence Interval (CI), 0.01, 0.45) and middle income (AOR:0.40,95% CI, 0.17, 0.97), parental/guardian education/no education (AOR:6.91, 95% CI, 2.52 8.93), marital status of the mother, being divorced(AOR:0.01 CI, 0.01, 0.05), leaving a child with another child for supervision [sometime (AOR:2.56,95% CI, 1.06, 6.20), and most often (AOR: 4.77, CI 1.15,9.77)] were factors statistically associated with RTI (Table 3).

**Table 3:** Simple and Multiple Logistic Regression Analysis of Factors Associated with RTI among Adolescents and Children in the Referral Hospitals of Amhara Regional State, Ethiopia. 2016 (*N* = 830)

Variables		RTI		Odds Ratio with 95% CI		*P*- Value
		Yes	No	COR	AOR	
Sex of the child	Male	113	395	1.45 (1.01 2.08)	1.76 (0.79 3.93	0.16
	Female	53	269	1	1	1
Guardian level of education	No	92	311	1.66 (1.04 2.67)	6.91 (2.52 8.93)	0.001*
	Primary	27	152	1.44 (0.89 2.34)	2.58 (0.85 7.82)	0.094
	Secondary	26	127	1.04 (0.61 1.78)	0.93 (0.18 4.65)	0.928
	Tertiary	21	74	1	1	1
Marital status of the mother	Single	13	43	1	1	1
	Married	130	524	1.22 (0.64 2.33)	0.28 (0.06 1.25)	0.095
	Separated	3	23	2.32 (0.59 8.97)	0.98 (0.07 12.95)	0.985
	Divorced	11	12	0.33 (0.12 0.92)	0.008 (0.01 0.05)	0.001*
	Widowed	9	62	2.083 (0.818 5.303)	0.51 (0.073 3.483)	0.489
Father’s Occupation	Farmer	74	311	1	1	
	Employed	36	199	1.31 (0.850 2.035)	2.803 (0.737 10.660)	0.130
	Petty trader	27	61	0.538 (0.32 0.90)	0.98 (0.21 4.55)	0.982
	Day laborer	29	93	0.76 (0.45 1.24)	3.12 (0.67 4.48)	0.147
leaving a child with a child	No	97	310	1	1	1
	sometimes	56	267	1.49 (1.03 2.15)	2.56 (1.06 6.20)	0.037*
	most often	13	87	2.09 (1.120 3.91)	4.77 (1.15 9.77)	0.031*
Child behavior	Aggressive	17	144	2.43 (1.42 4.14)	1.04 (0.33, 3.26)	0.944
	Unaggressive	149	520	1		
Socioeconomic status	Wealth	15	38	0.63 (0.32 1.23)	0.08 (0.01, 0.45)	0.004*
	Middle	97	409	1.05 (0.72 1.52)	0.41 (0.17,0.97)	0.044*
	Low	54	217	1	1	1
Age of Guardian	< 30	50	223	1	1	1
	31–40	68	263	0.87 (0.58 1.30)	0.72 (0.28 1.84)	0.492
	41–50	39	129	0.74 (0.46 1.19)	0.33 (0.12 1.02)	0.055
	51–65	9	49	1.22 (0.56 2.65)	1.66 (3.74 5.58)	0.001*

1: ref reference category, *COR* crude odds ratio, *AOR* adjusted odds ratio, * *p* < 0.05

## Discussion

This is the first study that has assessed the prevalence and factors associated with RTI among adolescents and children in the referral hospitals of Amhara National Regional State, Ethiopia. The overall prevalence of RTI was found to be 20%. This finding is higher than a study conducted in Iran, which documented a prevalence of 16.4% [12].The possible explanation of the differences between the two studies may be due to the variation of the countries in road development. Ethiopian is one of lest developed country, with poor road network [10], hence people can easily exposed to RTI at a higher level in Ethiopia somewhere else.

The current finding is also higher as compared to the study conducted in South Africa,13.3% [13], and in India,52.61% [14].The difference might be attributed to the study period, i.e. the South African and Indian studies were a three years study, while the current study is only a three months data analysis, in which case the result can be affected by seasonal variation of RTI occurrence.

Another study in India, on prevalence of RTI showed that the prevalence was 31% [15].The disparity may be due to study population differences, between these studies. For the Indian study, those children whose age was 18 were also included, while in the current study those children whose age was 18 were excluded. Therefore, the prevalence of Indian’s study may be higher than the current finding.

The probability of RTI for adolescents and children whose guardian or family were not educated was nearly 7 times more likely to have RTI as compared to those children whose guardian or family were educated at tertiary level (AOR: 6.91, 95% CI 2.52,8.93). Uneducated guardian or family may not have the required knowledge towards the prevention of such kind of injury. Thus, those children whose guardian or families were not educated can exposed to RTI more frequently.

The odds of RTI for children whose mother’s was divorced were 99.9% less likely to have RTI as compared to those children whose mother’s was single (AOR:0.01, CI 0.01,0.05). Although there was no study in line with this finding, the possible reason, might be in most of the Ethiopian community, following divorce, children of the divorced mother will joined their grand families, with the experiences of assisting children to grow in a well-protected manner. Thus, this practice can protect children from RTI.

In this study, leaving a child for supervision with another child is found to be a risk factor for the occurrence of RTI. The probability of RTI for children whose caregivers were leave a child with other child for supervision sometimes and most often were 2.5 and 4.7 times more likely to have RTI as compared to those children whose caregivers were not leave children with another child respectively. In Ethiopian there is an informal appointment of young’s before the age of 18 in order to supervised adolescents and school aged children. This practice could potentially expose for inadequate supervision of the children population, which can expose them for injury, such as RTI.

Furthermore, the odds of RTI for children whose families income described as wealthy and middle were 92 and 59.2% less likely to have RTI as compared to those children whose families’ income were described as low in that order,(AOR: 0.08, CI, 0.01, 0.45) and, (AOR:0.41, CI 0.17,0.97). Socioeconomic status of any family will have a determinant role in the health and development of children in general. The wellbeing of those children of a better income family tends to be protected and the probability of facing RTI will be lower for children from higher income families. This is the reflection of poverty on the health status of children and this is supported by a study done in Canada [16]. On the other hand, children from the families of wealthy will have a better environment for health and wellbeing in general.

Furthermore, the odds of RTI for children whose guardian age was between 51 and 65 years were 1.6 times more likely to have RTI as compared to those children whose guardian age was less than 30 years (AOR: 1.66, CI 3.74 5.58). This may be due to the effect of aging, as it decreases the ability of the old family members to provided adequate supervision for child’s well-being. Therefore, those children of the older age family may have RTI more frequently as compared to the children of the younger age families.

### Limitation of the study

In this study, the duration of the data collection was short so that the result may be affected by seasonal occurrences of RTI. Furthermore, this study was a hospital based study so it may not generalizable to the general population. Moreover, as the study design is cross-sectional it does not confirm a cause-and-effect relationship. Finally this study did not look other factors related to RTI, such as driver behavior, car condition and the road and its related infrastructure.

## Conclusion

In this study, the prevalence of RTI was found to be high. Socioeconomic status (wealthy and middle income), no education of guardian or family, mother’s marital status (divorced), and leaving a child with another child for sometimes and most often were factors independently associated with RTI. The finding suggest that RTI prevention needs a critical consideration and the intervention strategies shall focus on low income, uneducated and families who practice leaving a child with another child and further study is recommended to explore the relationship between divorced marital status of the mother and RTI.

## Abbreviations

AOR: Adjusted Odd Ratios
CI: Confidence Interval
ED: Emergency Department
IRB: Institutional Review Board
OR: Odd Ratios
RTI: Road Traffic Incident
